# Typhoid and Paratyphoid Cost of Illness in Pakistan: Patient and Health Facility Costs From the Surveillance for Enteric Fever in Asia Project II

**DOI:** 10.1093/cid/ciaa1336

**Published:** 2020-12-01

**Authors:** Nelly Mejia, Farah Qamar, Mohammad T Yousafzai, Jamal Raza, Denise O Garrett, Kashmira Date, Taiwo Abimbola, Sarah W Pallas

**Affiliations:** 1 Global Immunization Division, US Centers for Disease Control and Prevention, Atlanta, Georgia, USA; 2 Aga Khan University, Karachi, Pakistan; 3 National Institute of Child Health, Karachi, Pakistan; 4 Applied Epidemiology, Sabin Vaccine Institute, Washington, DC, USA

**Keywords:** typhoid, paratyphoid, enteric fever, cost of illness, Pakistan

## Abstract

**Background:**

The objective of this study was to estimate the cost of illness from enteric fever (typhoid and paratyphoid) at selected sites in Pakistan.

**Methods:**

We implemented a cost-of-illness study in 4 hospitals as part of the Surveillance for Enteric Fever in Asia Project (SEAP) II in Pakistan. From the patient and caregiver perspective, we collected direct medical, nonmedical, and indirect costs per case of enteric fever incurred since illness onset by phone after enrollment and 6 weeks later. From the health care provider perspective, we collected data on quantities and prices of resources used at 3 of the hospitals, to estimate the direct medical economic costs to treat a case of enteric fever. We collected costs in Pakistani rupees and converted them into 2018 US dollars. We multiplied the unit cost per procedure by the frequency of procedures in the surveillance case cohort to calculate the average cost per case.

**Results:**

We collected patient and caregiver information for 1029 patients with blood culture–confirmed enteric fever or with a nontraumatic terminal ileal perforation, with a median cost of illness per case of US $196.37 (IQR, US $72.89–496.40). The median direct medical and nonmedical costs represented 8.2% of the annual labor income. From the health care provider perspective, the estimated average direct medical cost per case was US $50.88 at Hospital A, US $52.24 at Hospital B, and US $11.73 at Hospital C.

**Conclusions:**

Enteric fever can impose a considerable economic burden in Pakistan. These new estimates of the cost of illness of enteric fever can improve evaluation and modeling of the costs and benefits of enteric fever prevention and control measures, including typhoid conjugate vaccines.

Enteric fever, an infection caused by *S*. Typhi or Paratyphi, is endemic in many low- and middle-income tropical countries among populations with low access to safe water and sanitation. Globally, in 2017 there were an estimated 10.9 million cases of typhoid fever and 3.4 million cases of paratyphoid fever [1]. The economic costs of any disease, including enteric fever, comprise the direct medical and nonmedical costs associated with seeking and receiving care, as well as the indirect costs of productivity loss due to illness or death, which are borne by households, health systems, and governments [2]. Growing trends of antibiotic resistance of *S.* Typhi and Paratyphi increase the economic costs of enteric fever by requiring more potent and expensive drugs for treatment [3].

Pakistan is a lower-middle income country with a population of 212 million [4]. Enteric fever is endemic in Pakistan, with an estimated incidence of 727.8 thousand cases and 8175 deaths in 2017 [5], with a growing trend of antibiotic-resistant enteric fever. Since November 2016, Pakistan has faced an outbreak caused by an extensively drug-resistant (XDR) typhoid strain (resistant to cotrimoxazole, ampicillin, chloramphenicol, and ceftriaxone, and resistant to or with reduced susceptibility to fluoroquinolones) [6]. There has been only 1 cost of illness (COI) study of typhoid fever in Pakistan; however, the sample size was small and purposive (n = 66 pediatric patients with a positive blood culture test for typhoid), and the data were collected in 2001 [7]. Similarly, there are COI studies of typhoid fever in other Asian countries (China, India, Indonesia, Nepal, and Vietnam) with data collected more than 17 years ago, between 1995 and 2003, and 1 in Nepal in 2015 that collected qualitative data [7–9]. These earlier studies might not reflect current health care and pricing structures in Pakistan. Moreover, except for the studies in Bangladesh and Nepal in 2018 from the Surveillance for Enteric Fever in Asia Project (SEAP) II, also included in this Supplement [10, 11], existent studies only included typhoid fever patients, excluding paratyphoid fever patients and those with severe illness, such as nontraumatic terminal ileal gastrointestinal perforations with no known etiology, if not lab confirmed; they also did not report information about specific treatment procedures for typhoid and paratyphoid.

This study aims to fill this gap in the evidence base for Pakistan. Results of these COI estimates can help evaluate investments in health policy interventions aiming to control and eliminate enteric fever, such as the introduction and scale-up of the new typhoid Vi-conjugate vaccine—recommended by the World Health Organization (WHO) and begun in Pakistan in 2019—and improvements in access to safe water and sanitation [6, 12]. Using data from SEAP II, we present detailed COI estimates at the procedure level for both typhoid and paratyphoid fever cases from 2 perspectives: (1) the patient and caregiver; and (2) the health care provider.

## METHODS

### Study Setting

This COI study was conducted as part of the economic component of SEAP II at 4 hospitals in urban areas of Karachi, in the Sindh Province of Pakistan. These health facilities included a public, not-for-profit pediatric hospital with teaching status; 2 private, not-for-profit teaching hospitals; and a public teaching hospital. These hospitals were purposively selected based on their laboratory capacity to perform blood culture testing for typhoid and paratyphoid, and they were not intended to be representative of health facilities at any geographical level in Pakistan.

### Cost of Illness From the Patient and Caregiver Perspective

#### Study Design

Patients were recruited in 3 hospitals between September 2016 and December 2018, and in a fourth hospital later added to the SEAP II study between January 2018 and December 2018. Eligible patients had a positive blood culture test for *S.* Typhi or Paratyphi or a nontraumatic terminal ileal perforation with no other known etiology, regardless of the blood culture result.

The COI evaluation from the patient and caregiver perspective included direct medical and nonmedical costs paid by any funding source, as well as indirect costs. Direct medical costs were defined as the monetary value of health facility registration fees, clinical examinations, inpatient stays, laboratory tests, drugs and medications, and other diagnostic and treatment services (eg, X-ray, surgery). Direct nonmedical costs were defined as the monetary value of transport, food, lodging, and care services for family members. Indirect costs were comprised of school days missed by patients and work time (including sick leave) lost by patients and caregivers (accompanying/providing care) due to the episode of enteric fever. School days missed were not monetized. Work productivity lost was only monetized for patients and caregivers ≥18 years at the median of the wage range reported (eg, if the monthly salary reported was in the range of 0–2400 Pakistani rupees, the midpoint of this range—1200 rupees—was used to value the work time). Costs excluded were the expenses on drugs, diagnostics, and treatments not related to enteric fever (eg, comorbidities and chronic conditions), goods and services that patients received at no charge, and intangible costs of pain and suffering.

#### Data Collection

Cost data questionnaires were designed in English and, after being piloted in the sites, were translated into Urdu. Bilingual SEAP II interviewers administered the questionnaires in Urdu and recorded the data electronically in a tablet. Cost data were collected at 2 time points by telephone: the first questionnaire was administered 2 to 3 days after a blood culture result or hospital discharge, if the patient received inpatient care; and the second questionnaire was administered 6 weeks (approximately 42 days) after study enrollment, at the same time as the SEAP II surveillance component follow-up call. The first questionnaire collected data on expenses incurred by the patient and caregivers from illness onset through the patient’s return home after the enrollment visit; the second questionnaire collected costs incurred after the enrollment visit until the follow-up call. Respondents were either patients ≥16 years or caregivers ≥18 years.

#### Cost of Illness Measures and Data Analysis

From the patient and caregiver perspective, COI measures included the median direct medical and nonmedical costs, median number of days of school lost by patients, median number of days of work lost and sick leave used by patients and/or caregivers, median wages lost by the patient and/or caregiver, and median total COI during the episode of enteric fever (sum of the direct medical costs, direct nonmedical costs, and indirect costs). For all COI measures, the median and the interquartile range were calculated.

The main analysis included COI measures for patients with enteric fever (typhoid and paratyphoid cases and nontraumatic terminal ileal perforation cases combined). In the main analysis, the value of the time spent by caregivers who did not regularly earn a wage (eg, unpaid household labor) only included the time spent at health facilities caring for or accompanying the patient, and was not monetized. In addition, as the value of the sick leave taken could have been borne by the employers and not by the employees, the main analysis also presented a COI estimate without the value of sick leave.

Sensitivity analyses were conducted to calculate COI measures separately for typhoid, paratyphoid, surgical, and nonsurgical cases, as well as those with multidrug-resistant (MDR) *S.* Typhi and *S.* Paratyphi (exclusively resistant to ampicillin, chloramphenicol, and cotrimoxazole) or XDR *S.* Typhi (XDR *S.* Paratyphi was excluded because there were no XDR cases). In addition, sensitivity analyses were conducted without outliers for each cost category (eg, examination fee, drugs, transportation). Outliers were defined as the observations with values above or below 2.24 standard deviations from the mean [13]. Other sensitivity analyses were conducted to estimate the indirect costs of patients and caregivers under different wage assumptions to address unpaid labor by (1) imputing their wage with the minimum daily wage rate of 675 rupees (or US $5.56 [14]); and (2) with the median wage reported by the respondents with paid labor. Finally, a sensitivity analysis to estimate the lifetime indirect costs of patients who died was also included. The value of a patient’s future earnings were calculated using the average life expectancy in Pakistan as reported by the WHO [15], and using the minimum wage rate as a proxy for income, adjusted by an annual inflation rate of 3.66% (the average annual inflation rate in Pakistan between 2016–2018 [16]) and discounted at a standard annual rate of 3% [2].

Costs were collected in Pakistani rupees, adjusted to 2018 values based on inflation rates and converted into 2018 US dollars using the annual average exchange rate for 2018 (121.48 rupees per US dollar [17]). Missing wage information for patients or caregivers was imputed with the median wage of the respondent sample.

### Cost of Illness From the Health Care Provider Perspective

#### Study Design

From the health care provider perspective, the COI was estimated as the direct medical economic costs (ie, the value of all resources used, not only financial outlays) incurred by the health facility to diagnose and treat a patient with enteric fever and its complications. The health facility sample included 3 of the 4 health facilities participating in the SEAP II surveillance and the patient and caregiver COI studies: a public, not-for-profit pediatric hospital with teaching status, and 2 private, not-for-profit teaching hospitals. The fourth hospital was added to SEAP II in January 2018, too late to be included in the health care provider perspective evaluation. Activity-based macro-costing was used to estimate the cost of procedures for which resource use per unit was assumed not to differ between patients with enteric fever and patients with other diseases (excluding medication costs)—for example, outpatient visits, inpatient bed days, emergency visits, pediatric intensive care unit bed days, intensive care unit bed days, outpatient surgery visits, inpatient surgery bed days—as well as to allocate the value of administrative services (eg, security, marketing, etc.), utilities and communications (eg, gas, electricity, water), and cross-cutting clinical supporting services (laundry, cleaning, etc.). Ingredients-based micro-costing was used to estimate the costs of specific procedures for which activity-based macro-costing by ward could not be conducted because the resource use per unit varied by procedure: blood draws, blood culture tests, complete blood counts, abdominal X-rays, abdominal ultrasounds, surgeries for intestinal perforation, and gallbladder surgeries.

Resource inputs included in the cost estimation were personnel time and salaries, materials and supplies, equipment and instruments, contracted services, and the equivalent rental value of the building space. Excluded costs were those associated with magnetic resonance imaging, computed tomography scans, Widal tests, and C-reactive protein tests (reportedly infrequently used for enteric fever diagnosis in the study sites); patient registration; medication costs paid for by patients (rather than by the hospital as part of procedure provision); costs unrelated to enteric fever (eg, anti-malarial treatment, comorbidities, chronic conditions); nonclinical costs (eg, teaching salaries and classroom space) for the medical-school aspects of these teaching hospital sites; evaluation-specific costs; and the value of the study team staff time for project management, technical assistance, and evaluation.

#### Data Collection

Data on prices and quantities of resources used, as well as service volumes, for the 2015–2016 fiscal year were collected using a Microsoft Excel tool that had been piloted in the sites. Data were collected between June 2017 and April 2018 from annual financial reports, administrative records, on-site observation, and interviews with administrative and medical staff by a local consultant economist and by local SEAP II study team staff, with technical assistance from US Centers for Disease Control and Prevention (CDC) staff. Missing prices of supplies and materials or equipment and instruments were imputed with data from the United Nations International Children’s Emergency Fund (UNICEF) supply catalog [18]; if prices were unavailable from UNICEF, they were imputed from the other SEAP II hospitals in Pakistan. Data on the frequencies of procedures conducted for the patients with blood culture–confirmed enteric fever or nontraumatic ileal perforation in these health facilities were collected through the SEAP II surveillance component during September 2016–December 2018.

#### Cost-of-Illness Measures and Data Analysis

Data were analyzed in Microsoft Excel. For procedure costs estimated using ingredients-based micro-costing (and for the personnel costs of general services not specific to enteric fever), the unit cost per clinical procedure was calculated as the sum of the products of the quantity of resources used in that procedure, multiplied by that resource’s price (or monetary value):

$$=\underset{j=1}{\sum^{N}}(quantity\;of\;resource\;input\;used_{ij}\text{*}price\;of\;resource\;input_{j})$$

Here, *i* is the procedure, and *j* indexes each resource input used in the procedure up to N resources.

Due to the lack of information disaggregated at the ward level, for procedure costs estimated using activity-based macro-costing, the monetary value of resources (eg, supplies and machinery), except personnel, used in that procedure over the fiscal year was calculated as the sum of the products of the quantity of resources used in the whole hospital, multiplied by that resource’s price (or monetary value) and then divided by the hospital’s service volume (total number of services). Thus, the value of resources was evenly allocated across all services:

$$=\;\frac{\underset{j=1}{\overset{N}{\sum }}(quantity\;of\;resource\;input\;used_{j}\text{*}price\;of\;resource\;input_{j})}{service\;volume}$$

Here, *j* indexes each resource input used in the hospital up to N resources.

As most information about administrative costs in the 3 hospitals was missing—namely, hospital-level cross-cutting utilities and communication services, administrative services, and clinical support services (eg, laundry, cleaning)—these costs were imputed. All procedure costs were increased by the proportion that those administrative costs represented as part of the nonadministrative costs by micro-costing or activity-based procedures across the 4 participating hospitals in SEAP II in Nepal and Bangladesh, which had more complete information. Thus, the estimated costs of procedures using ingredients-based micro-costing were increased by 36%, and the estimated costs of activity-based procedures were increased by 20%.

The average direct economic medical cost per case of enteric fever was calculated by multiplying the unit cost per clinical procedure by the procedure’s frequency in the patient cohort of enteric fever cases identified through blood culture confirmation or nontraumatic ileal perforation from the surveillance study component, regardless of their participation in the patient COI component. These costs were summed across all procedures, then divided by the number of enteric fever cases from the surveillance component:

$$=\frac{\sum_{k=1}^{K}(health\;facility\;unit\;cost\;procedure_{i}\text{*}frequency\;of\;procedure_{ik})}{Total\;number\;of\;confirmed\;enteric\;fever\;cases\;(K)}$$

Here, *i* is the procedure, *k* indexes the confirmed enteric fever cases at the health facility, and K is the total number of enteric fever cases identified at the health facility during the surveillance study period.

Health facility costs were collected in the local currency (Pakistani rupees), adjusted to 2018 values based on inflation rates from the Pakistan Bureau of Statistics [16], and converted into US dollars using the average exchange rate for 2018 (121.48 Pakistani rupees per US dollar [17]).

#### Ethical Considerations

The study protocol was approved by the Pakistan Ethical Review Committee. In accordance with the human subjects review procedures of the US CDC, it was determined that the CDC was not formally engaged in human subjects research.

## RESULTS

### Cost of Illness From the Patient and Caregiver Perspective

#### Patient Characteristics

The first cost questionnaire captured responses from 1029 eligible patients or their caregivers (Table 1). Of these, 746 responded to the second questionnaire, for a 72.5% response rate. Of the first-questionnaire respondents, 78.4% had a positive blood culture test for *S.* Typhi, 9.2% had a positive blood culture test for *S.* Paratyphi, and 12.3% had a nontraumatic terminal ileal perforation (regardless of blood culture results). Among those with a positive blood culture, 170 (18.8%) had MDR enteric fever and 401 (44.5%) had XDR typhoid fever. More than half were male (60.6%), and 10.4% were younger than 2 years old: 19.7% were 2 to 4 years old, 43.7% were 5 to 17 years old, and 26.1% were ≥18 years. Nearly all patients’ households had a household flush toilet (95.5%), electricity (95.6%), a cement roof (95.4%), and a mobile phone (94.5%), but only 21.6% reported treating their drinking water by boiling or other methods.

**Table 1.** Patient and Caregiver Cost of Illness Due to Enteric Fever, Sample Characteristics, Karachi, Pakistan, September 2016–December 2018

Characteristic	n	%
Respondents		
Patients responding to enrollment cost questionnaire	1029	100.0
Patients responding to 6-week follow-up cost questionnaire	746	72.5
Patients who died of enteric fever	1	0.1
Blood culture result		
*Salmonella* Typhi positive	807	78.4
*Salmonella* Paratyphi positive	95	9.2
Not positive for either *Salmonella* Typhi or Paratyphi (surgical cases)	127	12.3
Drug resistance of *Salmonella* Typhi or *Salmonella* Paratyphi positive cases, n = 902		
Multidrug-resistant^a^*Salmonella* Typhi or *Salmonella* Paratyphi positive	170	18.8
Extensively drug-resistant^b^*Salmonella* Typhi positive	401	44.5
No drug–resistant *Salmonella* Typhi or *Salmonella* Paratyphi positive	331	36.7
Age, years		
<2	107	10.4
2–4	203	19.7
5–17	450	43.7
18+	269	26.1
Sex		
Male	624	60.6
Female	405	39.4
Household with mobile phone		
Yes	972	94.5
No	30	2.9
Did not respond	27	2.6
Households with electricity		
Yes	984	95.6
No	16	1.6
Did not respond	29	2.8
Households with car/motorcycle		
Yes	676	65.7
No	326	31.7
Did not respond	27	2.6
Household roof material		
Cement	982	95.4
Metal sheets, mats, ceramic, shingles	27	2.6
Natural materials	20	1.9
Households with sanitation		
No toilet	7	0.7
Household flush to sewer system, septic tank, somewhere else	983	95.5
Household pit latrine, bucket or hanging toilet, communal toilet, other	12	1.2
Did not respond	27	2.6
Drinking water treated at home		
Boil	202	19.6
Chlorine liquid, powder, or tablets	15	1.5
Other	5	0.5
Do not treat water	616	59.9
Did not respond	191	18.6

^a^Patients with blood culture–confirmed *Salmonella* Typhi or *S.* Paratyphi resistant to ampicillin, chloramphenicol, and cotrimoxazole. It excludes extensively drug-resistant *S.* Typhi cases.

^b^Patients with blood culture–confirmed *S.* Typhi resistant to ampicillin, chloramphenicol, cotrimoxazole, or ceftriaxone, and resistant to or with reduced susceptibility to fluoroquinolones (ciprofloxacin, levofloxacin, gatifloxacin, moxifloxacin, or ofloxacin). *S.* Paratyphi excluded because there was no extensively drug-resistant case in the sample.

#### Patient and Caregiver Direct Medical and Nonmedical Costs

**Figure 1.**
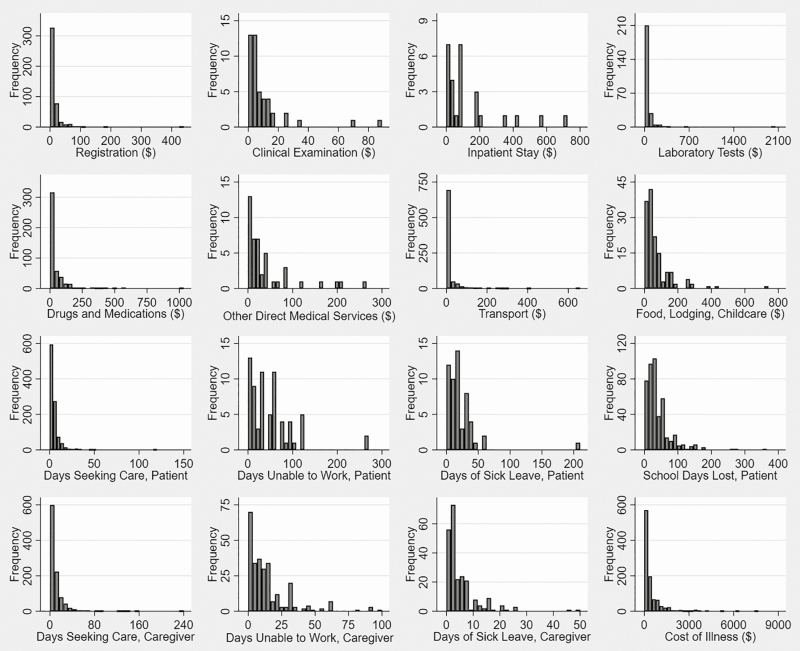
Distribution of cost-of-illness elements from the patient and caregiver perspective, Karachi, Pakistan, September 2016–December 2018. N = 1029 patients. Data are in 2018 US$.

Of the 1029 enteric fever patients, 980 (95.2%) reported direct medical costs, of whom 146 (14.2%) reported some inpatient care costs (this category includes patients who reported any inpatient care expense and all patients with nontraumatic ileal perforation; Table 2). Across all patients, the median direct medical cost was US $145.98 (interquartile range [IQR], US $53.51–$387.73). Although not all respondents were able to recall the costs they had paid for specific procedures, the largest median direct medical cost by procedure subcategory was inpatient stay (US $74.09; expenditure on bed days, not including any other diagnostic or treatment services), followed by laboratory results (US $25.66; Figure 1). The costs most frequently reported were drugs and medications (reported by 47.0%; median cost, US $16.46), registration (reported by 45.9%; median cost, US $4.12), and laboratory tests (reported by 25.9%; median cost, US $25.66). The median costs were higher in each category for patients reporting inpatient care than for patients with only outpatient care.

**Table 2.** Patient and Caregiver Cost of Illness Due to Enteric Fever: Direct Medical, Direct Nonmedical, Indirect, and Total Costs, Karachi, Pakistan, September 2016–December 2018

	Patients Who Only Reported Outpatient Care Expenses and did not Have a Nontraumatic Terminal Ileal Perforation^a^				Patients Who Reported Inpatient Care Expenses or Had a Nontraumatic Terminal Ileal Perforation^b^				All Patients			
Cost Type	n	Median	25^th^ Pctl	75^th^ Pctl	n	Median	25^th^ Pctl	75^th^ Pctl	n	Median	25^th^ Pctl	75^th^ Pctl
Direct medical costs, 2018 US$												
Total direct medical costs	834	126.27	47.04	374.56	146	235.85	115.25	456.06	980	145.98	53.51	387.73
Registration	399	4.12	2.14	17.11	51	4.28	1.71	16.46	450	4.12	2.06	16.46
Clinical examination	25	4.12	1.65	6.84	21	10.70	4.12	16.46	46	5.14	2.88	11.55
Inpatient stay	0	N/A	N/A	N/A	27	74.09	23.09	179.62	27	74.09	23.09	179.62
Laboratory tests	203	25.66	10.70	49.39	51	32.93	8.23	82.32	254	25.66	10.26	51.86
Drugs and medications	371	12.83	6.84	41.16	90	32.93	10.26	82.32	461	16.46	7.00	49.39
Other services^c^	8	8.42	3.34	13.26	36	25.18	10.45	48.57	44	23.04	8.23	41.16
Direct nonmedical costs, 2018 US$												
Total direct nonmedical costs	694	4.57	2.47	10.26	138	36.86	9.06	115.25	832	5.35	2.63	16.46
Transport	691	4.28	2.47	8.55	138	18.86	8.23	57.63	829	5.13	2.57	12.35
Food, lodging, childcare	76	32.93	16.46	82.32	68	49.39	31.28	90.55	144	41.16	21.38	83.93
Direct medical and nonmedical expenses, 2018 US$												
Direct medical and nonmedical preenrollment expenses	591	21.38	7.41	53.51	120	53.03	16.79	141.69	711	24.70	8.23	76.55
Total direct medical and nonmedical expenses	859	129.24	48.57	384.89	147	316.94	196.72	605.40	1006	162.59	57.79	419.84
Indirect costs: patient												
Days spent seeking care	868	1.32	0.13	4.00	149	9.00	6.00	15.00	1017	3.00	0.17	6.00
Days unable to work	27	14.00	4.00	48.00	42	58.50	30.00	85.00	69	35.00	14.00	60.00
Days of sick leave	51	14.00	7.00	28.00	4	40.00	15.00	135.00	55	15.00	7.00	28.00
School days lost	399	30.00	15.00	46.00	44	67.50	30.00	120.00	443	30.00	15.00	50.00
Total productivity loss, 2018 US$	74	96.30	46.38	198.61	42	327.71	173.91	498.07	116	147.43	68.79	287.99
Indirect costs: caregiver												
Days spent accompanying the patient seeking care	849	3.31	0.33	9.00	148	19.00	11.01	33.50	997	5.00	0.42	12.00
Days unable to work	167	5.00	2.00	12.00	107	15.00	8.00	30.00	274	9.00	3.00	17.00
Days of sick leave	217	3.00	1.33	6.00	14	6.50	3.00	20.00	231	3.00	2.00	6.00
Total productivity loss, 2018 US$	373	21.24	13.24	49.74	116	92.69	47.25	173.91	489	30.19	13.24	79.44
Cost of illness, 2018 US$												
Total cost of illness per case	863	149.11	60.23	424.31	148	476.12	250.26	983.85	1011	196.37	72.89	496.40
Total cost of illness per case without the value of sick leave	859	140.77	51.55	409.55	147	471.13	250.26	978.30	1006	175.15	64.15	484.11

N = 1029.

Abbreviations: N/A, not applicable because it excludes inpatient care; Pctl, percentile.

^a^ Expenses by patients who only reported outpatient care, regardless of patient recruitment location (outpatient care, inpatient care, hospital laboratory, surgery) and excluding patients with nontraumatic terminal ileal perforation.

^b^ Expenses by patients with nontraumatic terminal ileal perforation, regardless of whether they reported inpatient care, and patients reporting inpatient care expenses, regardless of patient recruitment location (outpatient care, inpatient care, hospital laboratory, surgery).

^c^ Other services that patients did not report in the above categories (eg, medical materials or equipment for surgery).

The median direct nonmedical cost was US $5.35 (IQR, US $2.63–$16.46), reported by 832 patients (80.9%; Table 2). Transport was the most frequently reported cost of this type (reported by 99.6% of those with any direct nonmedical costs; median cost, US $5.13), but the expenses on food, lodging, and childcare were the highest (median cost, US $41.16; reported by 17.3% of patients with direct nonmedical costs; Figure 1). The direct nonmedical costs of patients reporting inpatient care were higher in all cases than the costs of patients only reporting outpatient care.

More than two-thirds of the patients (69.1%) reported direct medical and nonmedical costs for seeking care at health facilities before enrollment in the study (Table 2), and 12.1% previously sought care at pharmacies. The median direct medical and nonmedical cost incurred by all patients before enrollment was US $24.70 (IQR, US $8.23–$76.55), which represents 15.2% of the median total direct medical and nonmedical costs incurred over the entire episode of enteric fever up to the second questionnaire administration date.

#### Patient and Caregiver Indirect Costs

From illness onset until the time of COI interview at 6 weeks (or up to the first questionnaire if the second questionnaire was not answered), a median of 3.00 days were spent across 1017 patients seeking and receiving care (IQR, 0.17–6.00 days; Table 2; Figure 1). A median of 30.00 days of school were lost (IQR, 15.00–50.00 days) across 443 patients. A median of 35.00 lost work days (IQR, 14.00–60.00 days) were reported across 69 patients, and a median of 15.00 sick leave days were used (IQR, 7.00–28.00 days) across 55 patients. On average, patients received care from 2 caregivers. Caregivers for 997 patients spent a median of 5.00 days (IQR, 0.42–12.00 days) accompanying the patient while seeking and receiving care. Caregivers for 274 patients were unable to work for a median of 9.00 days (IQR, 3.00–17.00 days); in addition, caregivers for 231 patients used a median of 3.00 days of sick leave (IQR, 2.00–6.00 days). When valued at the median of patients’ and caregivers’ reported wage ranges, days of work lost, plus sick leave, resulted in a median productivity loss of US $147.43 (IQR, US $68.79–$287.99) for patients and US $30.19 (IQR, US $13.24–$79.44) for caregivers. For patients who received inpatient care, these median indirect costs were higher for all categories than for patients only receiving outpatient care.

#### Median Cost Per Case of Enteric Fever

After adding direct medical costs, direct nonmedical costs, and indirect costs of patient and caregiver productivity losses, the median cost of illness per case of enteric fever from the patient and caregiver perspective was US $196.37 (IQR, US $72.89–$496.40) for all patients (Table 2; Figure 1). Patients receiving inpatient care reported a median COI 3 times higher than that of patients only reporting outpatient care. The median COI without the value of the sick leave days used decreased by 11%, to US $175.15 (IQR: US $64.15–$484.11).

#### Sensitivity Analysis

The results changed slightly when removing outlier values; the median COI decreased from US $196.37 to US $178.76 (Table 3). The median COI increased by 34% when imputing wages for unpaid labor (to US $264.45 for all patients) and by 31% when using the national minimum wage rates for unpaid labor of caregivers and patients (to US $256.46 for all patients; Table 4). When adding the future productivity losses of the 1 patient in the sample who died, evaluated at the minimum wage rate, the median COI does not change (US $196.37 for all patients; Table 4). When analyzing the COIs for typhoid (78.4%) and paratyphoid (9.2%) cases, the results are qualitatively similar, with higher costs of both the overall COI and most subcategories among patients with typhoid (median COI of US $165.98 for all patients) compared to patients with paratyphoid (median COI of US $127.57 for all patients; Tables 5 and 6). The COI results for patients with nontraumatic ileal perforation (12.3%) were higher overall (and in most subcategories of direct medical, nonmedical, and indirect costs) than for patients without these complications (blood culture–confirmed *S.* Typhi and Paratyphi cases), with an overall median COI of US $508.49, which is 3 times higher than for typhoid cases and 4 times higher than for paratyphoid cases (Tables 5–7). Given the high cost of surgical cases, when they are removed from the analysis the median COI for all patients (combined blood culture–confirmed *S.* Typhi and Paratyphi cases) decreases to US $155.73 (Table 7). The COI results for patients with MDR enteric fever (median COI, US $133.50) were lower than that for all patients in the sample (US $196.37), while the COI results for patients with XDR typhoid fever (median COI, US $251.34) were higher than that for all patients in the sample, but lower than for patients with nontraumatic ileal perforation (median COI, US $508.49; Tables 7–9).

**Table 3.** Sensitivity Analysis of Patient and Caregiver Cost of Illness Due to Enteric Fever: Direct Medical, Direct Nonmedical, Indirect, and Total Costs When Excluding Outliers, Karachi, Pakistan, September 2016–December 2018

	Patients Who Only Reported Outpatient Care Expenses and did not Have a Nontraumatic Terminal Ileal Perforation^a^				Patients Who Reported Inpatient Care Expenses or Had a Nontraumatic Terminal Ileal Perforation^b^				All Patients			
Cost Type	n	Median	25^th^ Pctl	75^th^ Pctl	n	Median	25^th^ Pctl	75^th^ Pctl	n	Median	25^th^ Pctl	75^th^ Pctl
Direct medical costs, 2018 US$												
Total direct medical costs	809	123.17	45.28	346.40	139	228.03	109.49	411.61	948	133.57	49.50	360.72
Registration	393	4.12	2.14	16.46	48	4.12	1.68	12.55	441	4.12	2.06	16.46
Clinical examination	25	4.12	1.65	6.84	19	5.76	4.12	15.40	44	4.70	2.76	10.29
Inpatient stay	0	N/A	N/A	N/A	25	74.09	23.09	90.55	25	74.09	23.09	90.55
Laboratory tests	202	25.66	10.70	49.39	50	32.11	8.23	82.32	252	25.66	10.07	51.32
Drugs and medications	359	12.35	6.59	34.21	88	29.29	10.07	82.32	447	14.82	6.84	42.77
Other services^c^	7	6.59	2.57	11.12	34	24.70	8.55	41.16	41	18.82	8.23	41.16
Direct nonmedical costs, 2018 US$												
Total direct nonmedical costs	683	4.28	2.47	9.88	120	23.87	8.23	75.96	803	5.13	2.57	13.17
Transport	682	4.28	2.47	8.55	126	16.46	8.23	45.28	808	4.94	2.57	11.28
Food, lodging, childcare	76	32.93	16.46	82.32	63	49.39	25.66	82.32	139	41.16	18.52	82.32
Indirect costs: patient												
Days spent seeking care	864	1.29	0.13	4.00	126	8.00	6.00	12.00	990	2.46	0.15	5.00
Days unable to work	27	14.00	4.00	48.00	40	53.50	30.00	80.00	67	30.00	14.00	60.00
Days of sick leave	51	14.00	7.00	28.00	3	20.00	10.00	60.01	54	14.50	7.00	28.00
School days lost	392	30.00	15.00	45.00	35	58.00	22.00	90.00	427	30.00	15.00	50.00
Total productivity loss, 2018 US$	74	96.30	46.38	198.61	40	311.16	161.56	479.98	114	141.61	68.79	275.14
Indirect costs: caregiver												
Days spent accompanying the patient seeking care	846	3.26	0.33	9.00	125	16.00	10.13	24.00	971	4.31	0.42	11.00
Days unable to work	164	5.00	2.00	12.00	96	15.00	8.00	22.00	260	8.00	3.00	15.00
Days of sick leave	211	3.00	1.17	5.00	10	4.50	2.00	7.00	221	3.00	1.33	5.00
Total productivity loss, 2018 US$	368	20.69	13.24	46.34	102	82.75	41.34	127.53	470	27.51	13.24	68.79
Cost of illness												
Total cost of illness per case, 2018 US$	838	141.68	57.63	397.91	137	460.90	244.91	833.13	975	178.76	68.87	455.89

Abbreviations: N/A, not applicable because it excludes inpatient care; Pctl, percentile.

^a^ Expenses by patients who only reported outpatient care, regardless of patient recruitment location (outpatient care, inpatient care, hospital laboratory, surgery) and excluding patients with nontraumatic terminal ileal perforation.

^b^ Expenses by patients with nontraumatic terminal ileal perforation, regardless of whether they reported inpatient care, and patients reporting inpatient care expenses, regardless of patient recruitment location (outpatient care, inpatient care, hospital laboratory, surgery).

^c^ Other services that patients did not report in the above categories (eg, medical materials or equipment for surgery).

**Table 4.** Sensitivity Analysis of Patient and Caregiver Cost of Illness Due to Enteric Fever: Indirect and Total Costs When Using Alternative Wage Rates and Including Unpaid Labor, Karachi, Pakistan, September 2016–December 2018

	Patients Who Only Reported Outpatient Care Expenses and did not Have a Nontraumatic Terminal Ileal Perforation^a^				Patients Who Reported Inpatient Care Expenses or Had a Nontraumatic Terminal Ileal Perforation^b^				All Patients			
Cost Type	n	Median	25^th^ Pctl	75^th^ Pctl	n	Median	25^th^ Pctl	75^th^ Pctl	n	Median	25^th^ Pctl	75^th^ Pctl
Indirect costs: imputing median wage in the sample for unpaid patient and caregivers’ time												
Total productivity loss	871	54.07	6.85	113.10	149	317.78	167.16	557.77	1020	72.82	12.83	164.94
Cost of illness: imputing median wage in the sample for unpaid patient and caregivers’ time												
Total cost of illness per case	872	197.58	82.77	510.11	150	666.33	411.41	1327.97	1022	264.45	97.73	616.53
Indirect costs: imputing country minimum wage for unpaid patient and caregivers’ time												
Total productivity loss	871	48.16	5.90	100.01	149	292.25	150.03	532.92	1020	63.42	11.51	148.99
Cost of illness: imputing country minimum wage for unpaid patient and caregivers’ time												
Total cost of illness per case	872	186.25	79.60	495.78	150	643.43	389.38	1267.99	1022	252.23	93.19	602.37
Indirect costs: imputing country minimum wage for future productivity losses of 1 patient who died												
Total productivity loss	425	27.51	13.24	72.82	124	125.15	50.57	372.52	549	39.72	13.76	99.31
Cost of illness: imputing country minimum wage for future productivity losses of 1 patient who died												
Total cost of illness per case	863	149.11	60.23	424.31	148	476.12	250.26	992.31	1011	196.37	72.89	498.04

N = 1029. Data are shown in 2018 US$.

Abbreviation: Pctl, percentile.

^a^ Expenses by patients who only reported outpatient care, regardless of patient recruitment location (outpatient care, inpatient care, hospital laboratory, surgery) and excluding patients with nontraumatic terminal ileal perforation.

^b^ Expenses by patients with nontraumatic terminal ileal perforation, regardless of whether they reported inpatient care, and patients reporting inpatient care expenses, regardless of patient recruitment location (outpatient care, inpatient care, hospital laboratory, surgery).

**Table 5.** Patient and Caregiver Cost of Illness Due to Typhoid Fever: Direct Medical, Direct Nonmedical, Indirect, and Total Costs, Karachi, Pakistan, September 2016–December 2018

	Patients Who Only Reported Outpatient Care Expenses and did not Have a Nontraumatic Terminal Ileal Perforation^a^				Patients Who Reported Inpatient Care Expenses or Had a Nontraumatic Terminal Ileal Perforation^b^				All Patients			
Cost Type	n	Median	25^th^ Pctl	75^th^ Pctl	n	Median	25^th^ Pctl	75^th^ Pctl	n	Median	25^th^ Pctl	75^th^ Pctl
Direct medical costs, 2018 US$												
Total direct medical costs	754	131.71	47.04	384.89	23	299.36	203.33	576.25	777	137.07	49.39	395.14
Registration	350	4.12	1.73	17.11	16	16.46	8.23	43.63	366	4.12	2.06	17.11
Clinical examination	24	4.12	1.44	7.13	7	15.40	2.88	24.70	31	4.12	1.65	9.06
Inpatient stay	0	N/A	N/A	N/A	23	82.32	32.93	185.60	23	82.32	32.93	185.60
Laboratory tests	170	24.16	9.88	44.48	13	56.45	12.35	82.32	183	24.70	9.88	49.39
Drugs and medications	327	13.17	6.84	41.16	19	82.32	24.70	118.54	346	14.26	6.84	49.39
Other services^c^	6	8.85	4.12	15.40	2	18.41	5.77	31.05	8	8.85	4.94	23.22
Direct nonmedical costs, 2018 US$												
Total direct nonmedical costs	627	4.94	2.47	10.26	20	4.20	2.84	19.55	647	4.77	2.47	10.26
Transport	624	4.28	2.47	8.73	20	4.12	2.84	10.29	644	4.28	2.47	8.73
Food, lodging, childcare	69	32.93	16.46	82.32	5	18.52	9.88	34.21	74	32.93	16.46	82.32
Indirect costs: patient												
Days spent seeking care	775	2.00	0.13	4.42	23	4.00	0.21	6.00	798	2.00	0.13	5.00
Days unable to work	20	19.50	8.00	60.00	0	…	…	…	20	19.50	8.00	60.00
Days of sick leave	31	15.00	7.00	30.00	0	…	…	…	31	15.00	7.00	30.00
School days lost	356	30.00	16.50	50.00	4	29.00	20.00	33.50	360	30.00	16.50	48.50
Total productivity loss, 2018 US$	50	101.24	48.15	206.36	0	…	…	…	50	101.24	48.15	206.36
Indirect costs: caregiver												
Days spent accompanying the patient seeking care	765	4.00	0.33	9.13	23	6.33	2.00	10.13	788	4.00	0.33	9.14
Days unable to work	158	5.00	2.00	12.00	8	4.50	2.50	8.00	166	5.00	2.00	12.00
Days of sick leave	191	3.00	2.00	6.00	7	5.00	3.00	15.00	198	3.00	2.00	7.00
Total productivity loss, 2018 US$	339	26.48	13.24	52.96	14	33.75	23.19	59.58	353	26.48	13.24	52.96
Cost of illness, 2018 US$												
Total cost of illness per case	773	157.32	60.23	434.54	23	313.34	232.39	626.03	796	165.98	61.63	450.44

N = 807.

Abbreviations: N/A, not applicable because it excludes inpatient care; Pctl, percentile.

^a^ Expenses by patients who only reported outpatient care, regardless of patient recruitment location (outpatient care, inpatient care, hospital laboratory, surgery) and excluding patients with nontraumatic terminal ileal perforation.

^b^ Expenses by patients with nontraumatic terminal ileal perforation, regardless of whether they reported inpatient care, and patients reporting inpatient care expenses, regardless of patient recruitment location (outpatient care, inpatient care, hospital laboratory, surgery).

^c^ Other services that patients did not report in the above categories (eg, medical materials or equipment for surgery).

**Table 6.** Patient and Caregiver Cost of Illness Due to Paratyphoid Fever: Direct Medical, Direct Nonmedical, Indirect, and Total Costs, Karachi, Pakistan, September 2016–December 2018

	Patients Who Only Reported Outpatient Care Expenses and did not Have a Nontraumatic Terminal Ileal Perforation^a^				Patients Who Reported Inpatient Care Expenses or Had a Nontraumatic Terminal Ileal Perforation^b^				All Patients			
Cost Type	n	Median	25^th^ Pctl	75^th^ Pctl	n	Median	25^th^ Pctl	75^th^ Pctl	n	Median	25^th^ Pctl	75^th^ Pctl
Direct medical costs, 2018 US$												
Total direct medical costs	80	100.71	48.22	213.40	2	273.70	218.11	329.30	82	104.35	49.39	218.11
Registration	49	5.56	3.42	17.96	1	2.57	2.57	2.57	50	5.35	3.42	17.96
Clinical examination	1	5.56	5.56	5.56	0	…	…	…	1	5.56	5.56	5.56
Inpatient stay	0	N/A	N/A	N/A	2	7.27	5.13	9.41	2	7.27	5.13	9.41
Laboratory tests	33	42.77	20.53	64.15	1	145.40	145.40	145.40	34	43.19	20.53	68.43
Drugs and medications	44	11.31	5.52	25.18	1	8.55	8.55	8.55	45	10.26	5.56	24.70
Other services^c^	2	6.41	2.57	10.26	0	…	…	…	2	6.41	2.57	10.26
Direct nonmedical costs, 2018 US$												
Total direct nonmedical costs	67	4.28	2.05	11.97	2	14.11	12.83	15.40	69	4.28	2.14	11.97
Transport	67	4.28	2.05	8.55	2	14.11	12.83	15.40	69	4.28	2.14	9.06
Food, lodging, childcare	7	29.64	17.11	68.43	0	…	…	…	7	29.64	17.11	68.43
Indirect costs: patient												
Days spent seeking care	93	1.15	0.25	2.33	2	2.50	1.00	4.00	95	1.15	0.25	3.00
Days unable to work	7	3.21	3.00	12.00	1	2.00	2.00	2.00	8	3.10	2.50	8.50
Days of sick leave	20	14.00	6.50	22.00	0	…	…	…	20	14.00	6.50	22.00
School days lost	43	14.00	7.00	30.00	1	10.00	10.00	10.00	44	13.00	7.50	30.00
Total productivity loss, 2018 US$	24	87.61	35.57	134.99	1	8.62	8.62	8.62	25	82.54	28.98	132.41
Indirect costs: caregiver												
Days spent accompanying the patient seeking care	84	2.29	1.00	4.73	2	13.63	8.00	19.25	86	2.31	1.00	5.00
Days unable to work	9	7.00	2.00	14.00	1	1.00	1.00	1.00	10	4.50	1.00	14.00
Days of sick leave	26	2.00	1.13	4.00	0	…	…	…	26	2.00	1.13	4.00
Total productivity loss, 2018 US$	34	13.76	6.88	27.51	1	6.88	6.88	6.88	35	13.76	6.88	27.51
Cost of illness, 2018 US$												
Total cost of illness per case	90	126.11	64.15	266.86	2	295.57	239.56	351.57	92	127.57	64.59	267.98

N = 95.

Abbreviations: N/A, not applicable because it excludes inpatient care; Pctl, percentile.

^a^ Expenses by patients who only reported outpatient care, regardless of patient recruitment location (outpatient care, inpatient care, hospital laboratory, surgery) and excluding patients with nontraumatic terminal ileal perforation.

^b^ Expenses by patients with nontraumatic terminal ileal perforation, regardless of whether they reported inpatient care, and patients reporting inpatient care expenses, regardless of patient recruitment location (outpatient care, inpatient care, hospital laboratory, surgery).

^c^ Other services that patients did not report in the above categories (eg, medical materials or equipment for surgery).

**Table 7.** Patient and Caregiver Cost of Illness Due to Patients with Nontraumatic Terminal Ileal Perforation and Due to Patients Without Nontraumatic Terminal Ileal Perforation: Direct Medical, Direct Nonmedical, Indirect, and Total Costs, Karachi, Pakistan, September 2016–December 2018

	All Patients^a^ With Nontraumatic Terminal Ileal Perforation, n = 127				All Patients Without Nontraumatic Terminal Ileal Perforations, n = 902			
Cost Type	n	Median	25^th^ Pctl	75^th^ Pctl	n	Median	25^th^ Pctl	75^th^ Pctl
Direct medical costs, 2018 US$								
Total direct medical costs	121	228.03	102.64	419.84	859	128.30	49.39	379.91
Registration	34	2.88	0.86	5.56	416	4.12	2.18	17.20
Clinical examination	14	8.23	4.28	13.99	32	4.12	2.14	8.64
Inpatient stay	2	21.82	2.47	41.16	25	74.09	24.70	179.62
Laboratory tests	37	23.95	8.23	65.86	217	25.66	10.78	51.32
Drugs and medications	70	24.70	8.23	68.43	391	13.69	6.84	43.63
Other services^b^	34	25.18	12.35	55.98	10	8.42	4.12	15.40
Direct nonmedical costs, 2018 US$								
Total direct nonmedical costs	116	55.57	13.17	132.33	716	4.57	2.47	10.26
Transport	116	24.70	9.06	60.92	713	4.28	2.47	8.91
Food, lodging, childcare	63	49.39	32.93	94.67	716	4.57	2.47	10.26
Indirect costs: patient								
Days spent seeking care	124	10.00	7.00	16.50	893	1.46	0.13	4.00
Days unable to work	41	60.00	30.00	85.00	28	13.00	4.00	39.00
Days of sick leave	4	40.00	15.00	135.00	51	14.00	7.00	28.00
School days lost	39	90.00	50.00	120.00	404	30.00	15.00	45.50
Total productivity loss, 2018 US$	41	331.02	185.37	498.07	75	96.30	42.16	198.61
Indirect costs: caregiver								
Days spent accompanying the patient seeking care	123	21.50	14.00	36.17	874	3.44	0.33	9.00
Days unable to work	98	16.50	10.00	30.00	176	5.00	2.00	12.00
Days of sick leave	7	7.00	2.00	22.00	224	3.00	2.00	6.00
Total productivity loss, 2018 US$	101	103.76	62.26	198.61	388	23.19	13.24	49.95
Cost of illness, 2018 US$								
Total cost of illness per case	123	508.49	265.51	1068.14	888	155.73	61.74	430.37

Abbreviation: Pctl, percentile.

^a^ All patients with nontraumatic ileal perforation (regardless of whether they reported inpatient care expenses) are considered in the inpatient care category.

^b^ Other services that patients did not report in the above categories (eg, medical materials or equipment for surgery).

**Table 8.** Patient and Caregiver Cost of Illness Due to Multidrug-resistant Enteric Fever: Direct Medical, Direct Nonmedical, Indirect, and Total Costs, Karachi, Pakistan, September 2016–December 2018

	Patients Who Only Reported Outpatient Care Expenses and did Not Have a Nontraumatic Terminal Ileal Perforation^a^				Patients Who Reported Inpatient Care Expenses or Had a Nontraumatic Terminal Ileal Perforation^b^				All Patients			
Cost Type	n	Median	25^th^ Pctl	75^th^ Pctl	n	Median	25^th^ Pctl	75^th^ Pctl	n	Median	25^th^ Pctl	75^th^ Pctl
Direct medical costs, 2018 US$												
Total direct medical costs	159	91.95	34.21	295.53	5	299.36	246.96	427.66	164	106.29	40.11	316.38
Registration	84	4.12	1.72	22.22	4	35.68	10.29	68.00	88	4.12	1.93	23.46
Clinical examination	4	2.10	1.23	6.01	1	2.88	2.88	2.88	5	2.88	1.23	2.96
Inpatient stay	0	N/A	N/A	N/A	5	82.32	74.09	171.92	5	82.32	74.09	171.92
Laboratory tests	47	24.70	4.94	51.32	4	73.24	33.31	96.76	51	25.66	4.94	55.85
Drugs and medications	69	9.41	6.84	26.35	4	83.93	65.11	102.04	73	11.53	6.84	34.21
Other services^c^	2	134.84	4.12	265.56	0	…	…	…	2	134.84	4.12	265.56
Direct nonmedical costs, 2018 US$												
Total direct nonmedical costs	132	4.28	2.57	8.55	3	4.28	3.29	7.41	135	4.28	2.57	8.55
Transport	130	4.28	2.57	8.55	3	4.28	3.29	7.41	133	4.28	2.57	8.55
Food, lodging, childcare	8	25.66	3.29	41.80	0	…	…	…	8	25.66	3.29	41.80
Indirect costs: patient												
Days spent seeking care	163	1.08	0.13	3.00	5	6.00	4.00	6.00	168	1.19	0.13	3.19
Days unable to work	5	19.00	17.00	20.00	0	…	…	…	5	19.00	17.00	20.00
Days of sick leave	4	24.13	9.13	38.50	0	…	…	…	4	24.13	9.13	38.50
School days lost	84	21.50	13.00	37.00	2	29.00	28.00	30.00	86	23.00	13.00	37.00
Total productivity loss, 2018 US$	9	130.69	73.31	206.36	0	…	…	…	9	130.69	73.31	206.36
Indirect costs: caregiver												
Days spent accompanying the patient seeking care	161	2.58	0.29	7.00	5	8.00	6.33	8.23	166	3.01	0.31	7.15
Days unable to work	24	4.00	2.00	7.00	2	2.50	1.00	4.00	26	4.00	2.00	7.00
Days of sick leave	47	2.00	1.00	4.00	1	15.00	15.00	15.00	48	2.00	1.00	4.00
Total productivity loss, 2018 US$	69	13.76	6.88	33.10	3	26.48	6.88	99.31	72	13.76	6.88	33.38
Cost of illness, 2018 US$												
Total cost of illness per case	164	128.56	41.54	336.69	5	310.52	250.26	427.66	169	133.50	43.88	337.52

N = 170. Multidrug-resistant patients were defined as those with blood culture–confirmed *Salmonella* Typhi or *S.* Paratyphi exclusively resistant to ampicillin, chloramphenicol, and cotrimoxazole. It excludes extensively drug-resistant *S.* Typhi cases.

Abbreviations: N/A, not applicable because it excludes inpatient care; Pctl, percentile.

^a^ Expenses by patients who only reported outpatient care, regardless of patient recruitment location (outpatient care, inpatient care, hospital laboratory, surgery) and excluding patients with nontraumatic terminal ileal perforation.

^b^ Expenses by patients with nontraumatic terminal ileal perforation, regardless of whether they reported inpatient care, and patients reporting inpatient care expenses, regardless of patient recruitment location (outpatient care, inpatient care, hospital laboratory, surgery).

^c^ Other services that patients did not report in the above categories (eg, medical materials or equipment for surgery).

**Table 9.** Patient and Caregiver Cost of Illness Due to Extensively Drug-resistant Typhoid Fever: Direct Medical, Direct Nonmedical, Indirect, and Total Costs, Karachi, Pakistan, September 2016–December 2018

	Patients Who Only Reported Outpatient Care Expenses and did not Have a Nontraumatic Terminal Ileal Perforation^a^				Patients Who Reported Inpatient Care Expenses or Had a Nontraumatic Terminal Ileal Perforation^b^				All Patients			
Cost Type	n	Median	25^th^ Pctl	75^th^ Pctl	n	Median	25^th^ Pctl	75^th^ Pctl	n	Median	25^th^ Pctl	75^th^ Pctl
Direct medical costs, 2018 US$												
Total direct medical costs	375	205.8	71.62	670.92	12	210.33	107.84	312.82	387	205.80	71.62	658.57
Registration	148	4.12	1.65	12.35	6	12.35	4.94	41.16	154	4.12	1.71	12.35
Clinical examination	12	3.50	1.23	5.15	4	10.29	2.68	20.58	16	3.91	1.23	6.38
Inpatient stay	0	N/A	N/A	N/A	12	45.28	16.46	82.32	12	45.28	16.46	82.32
Laboratory tests	66	22.23	8.23	43.63	6	18.52	3.70	41.16	72	22.23	8.23	42.40
Drugs and medications	149	18.93	6.84	65.00	11	82.32	16.46	164.64	160	23.05	7.20	74.91
Other services^c^	1	11.12	11.12	11.12	0	…	…	…	1	11.12	11.12	11.12
Direct nonmedical costs, 2018 US$												
Total direct nonmedical costs	312	4.94	2.47	10.70	11	4.94	3.29	39.51	323	4.94	2.47	10.87
Transport	312	4.12	2.47	8.55	11	4.12	3.29	13.17	323	4.12	2.47	8.55
Food, lodging, childcare	43	34.21	16.46	82.32	5	18.52	9.88	34.21	48	33.57	16.46	82.32
Indirect costs: patient												
Days spent seeking care	385	3.00	0.13	6.00	12	4.50	0.10	7.00	397	3.00	0.13	6.00
Days unable to work	11	48.00	8.00	60.00	0	…	…	…	11	48.00	8.00	60.00
Days of sick leave	11	22.00	15.00	30.00	0	…	…	…	11	22.00	15.00	30.00
School days lost	159	35.00	25.00	55.00	1	37.00	37.00	37.00	160	35.00	25.00	55.00
Total productivity loss, 2018 US$	22	179.64	92.69	264.81	0	…	…	…	22	179.64	92.69	264.81
Indirect costs: caregiver												
Days spent accompanying the patient seeking care	382	6.06	0.42	12.06	12	4.60	0.40	13.56	394	6.04	0.42	12.08
Days unable to work	92	7.00	3.00	14.00	3	9.00	5.00	15.00	95	7.00	3.00	14.00
Days of sick leave	84	4.00	2.00	7.00	3	7.00	4.00	26.00	87	4.00	2.00	7.00
Total productivity loss, 2018 US$	174	29.58	13.76	66.20	6	52.96	33.10	99.31	180	33.10	17.73	66.20
Cost of illness, 2018 US$												
Total cost of illness per case	382	252.58	82.39	764.16	12	246.55	160.73	387.12	394	251.34	82.82	729.56

N = 401. Extensively drug-resistant patients were defined as those with blood culture–confirmed *Salmonella* Typhi resistant to ampicillin, chloramphenicol, cotrimoxazole, and ceftriaxone, and resistant or with reduced susceptibility to fluoroquinolones.

Abbreviations: N/A, not applicable because it excludes inpatient care; Pctl, percentile.

^a^ Expenses by patients who only reported outpatient care, regardless of patient recruitment location (outpatient care, inpatient care, hospital laboratory, surgery) and excluding patients with nontraumatic terminal ileal perforation.

^b^ Expenses by patients with nontraumatic terminal ileal perforation, regardless of whether they reported inpatient care, and patients reporting inpatient care expenses, regardless of patient recruitment location (outpatient care, inpatient care, hospital laboratory, surgery).

^c^ Other services that patients did not report in the above categories (eg, medical materials or equipment for surgery).

### Cost of Illness From the Health Care Provider Perspective

The average direct medical costs per case of enteric fever from the provider’s perspective were US $50.88 for Hospital A, US $52.24 for Hospital B, and US $11.73 for Hospital C (Hospital C includes only the costs of medical services specific to enteric fever; Table 10). The costliest procedures at Hospital A were a pediatric intensive care unit bed day (US $256.92), followed by an inpatient hospital bed day (US $71.75) and gallbladder surgery (US $64.76). The least costly procedures were a blood draw (US $1.42) and abdominal ultrasound (US $2.14). In Hospital B, the most expensive procedures were an intensive care unit bed day (US $162.24), an emergency visit (US $35.43), and an inpatient hospital bed day (US $12.36), while the least costly were a blood draw (US $1.98) and an abdominal X-ray (US $4.93). In Hospital C, the most expensive procedures were surgery for intestinal perforation (US $151.28) and gallbladder surgery (US $107.41), while the least costly was a blood draw (US $0.94). Data on the frequencies of some specific procedures were not available from the surveillance component.

**Table 10.** Health Care Provider Cost of Illness Due to Enteric Fever: Procedure Unit Costs and Frequencies, and Average Cost Per Case of Enteric Fever, Karachi, Pakistan, July 2015–June 2016

	Unit cost in 2018 US$			Frequency		
Procedure	Hospital A^a^	Hospital B^a^	Hospital C^a^	Hospital A^a^	Hospital B^a^	Hospital C^a^
General services not specific to enteric fever						
Pediatric intensive care unit, per patient, per day	$256.92	N/A	N/A	...b	N/A	N/A
Intensive care unit, per patient, per day	N/A	$162.24	...b	N/A	...b	...b
Inpatient hospital cost, per patient, per day	$71.75	$12.36	...b	0	1165	590
Surgical inpatient, per patient, per day	$44.15	$11.98	...b	0	0	4
Surgical outpatient visit, per patient, per visit	$6.52	$12.08	...b	22	1	1
Emergency, per patient, per visit	$5.23	$35.43	...b	...b	...b	...b
Outpatient routine service cost, per patient, per visit	$4.33	$21.18	...b	0	168	231
Services specific to enteric fever						
Gallbladder surgery	$64.76	...b	$107.41	...b	...b	...b
Surgery for intestinal perforation	$40.58	...b	$151.28	73	17	1
Complete blood count	...b	...b	...b	21	418	115
Blood culture	$23.28	$11.76	$10.25	22	497	365
Abdominal X-ray^c^	$3.60	$4.93	$3.80	11	123	8
Abdominal ultrasound	$2.14	$7.07	$2.11	12	84	7
Blood draw	$1.42	$1.98	$0.94	22	478	365
Total blood culture–confirmed enteric fever or nontraumatic ileal perforation cases	…	…	…	73	497	365
Weighted average cost per case by enrollment site	$50.88	$52.24	$11.73	…	…	…

N = 935.

Abbreviations: N/A, not applicable because this hospital does not offer that service.

^a^ Hospital A is a public-sector, not-for-profit pediatric hospital with teaching status; Hospitals B and C are private, not-for-profit teaching hospitals.

^b^ Missing information.

^c^ Based on number of chest X-rays as a proxy, as number of abdominal X-rays was not collected in the Surveillance for Enteric Fever in Asia Project (SEAP) II clinical surveillance component.

## DISCUSSION

This study found that costs related to enteric fever were considerable among the sample of patients in Pakistan, particularly among those with severe complications (surgical and XDR typhoid cases). Compared with the existing literature on patient and caregiver COI estimates, this study found direct medical and nonmedical costs that were almost 6 times higher than the previous published estimates for Pakistan (Table 11) [7–9, 19]. Similarly, within our SEAP country studies, the direct medical and nonmedical COI for Pakistan was higher when compared with Bangladesh and Nepal (Table 11) [10, 11]. Although this study found average indirect costs higher than those previously available for Pakistan, these were lower than the indirect costs in studies conducted in Tanzania and Indonesia. The higher direct medical and nonmedical costs found here reflect differences in the structures of the health care systems and organizations included in each study, as well as potentially higher health care prices at the time of this study versus when data were collected for most previous studies in 1995–2003, even after adjusting for inflation. The lower indirect costs in this study might be due to differences in the definition of indirect costs; specifically, this study only monetized the productivity losses related to work time and sick leave of patients and caregivers ≥18 years, whereas other studies monetized productivity losses due to work time, sick leave, and school days of all ages, including those <18 years.

The median direct medical costs for all patients and caregivers in our sample, for patients reporting inpatient care costs, and for patients reporting only outpatient costs were 340.7%, 550.4%, and 294.7%, respectively, of Pakistan’s all-source health expenditure per capita of US $42.85 (2016 value in 2018 US dollars [4]). This illustrates the considerable economic burden posed by enteric fever in this country. Compared with the annualized median wage rates reported in the sample of US $1906.67, the median direct medical and nonmedical costs per case represented approximately 8.2%, 15.6%, 15.5%, 5.6%, and 10.8% of the annual individual labor income for all patients, for patients reporting inpatient care costs, for patients with nontraumatic ileal perforation, for patients with MDR enteric fever, and for patients with XDR typhoid fever, respectively. The literature defines catastrophic health expenditures as COIs that exceed 10% of the annual household income [20–22]. The estimated direct medical and nonmedical costs per case of enteric fever estimated by this study for patients receiving inpatient care would be considered catastrophic if the patients and caregivers who reported productivity losses were the only income earners in their households (household income was not measured in this COI study).

**Table 11.** Average Costs for a Case of Enteric Fever for Children and Adults in Pakistan and Other Countries and Study Characteristics

	SEAP			Other Studies							
	Pakistan	Nepal [11]	Bangladesh [10]^a^	Nepal [9]	India [8]	Tanzania [19]^b^	Vietnam [7]^c^	China [7]^c^	Indonesia [7]	Pakistan [7]^d^	India [7]
All patients											
Direct (medical and nonmedical) ($)	$383.46	$111.72		$101.46	$55.11	$30.27	$59.34	$175.78	$88.83	$64.98	$9.82
Indirect (days of work/sick leave/school lost)	24.42	14.29		22							
Indirect cost of patients and caregivers ($)	$56.34	$21.02		$35.29	$48.02	$146.55	$8.99	$45.25	$65.53	$13.00	$8.18
Adults (≥18 years)											
Direct (medical and non-medical) ($)	$441.43	$107.25						$226.25	$163.10		
Indirect (days of work/sick leave/school lost)	30.80	10.75									
Indirect cost of patients and caregivers ($)	$145.35	$28.38				$128.28		$62.65	$125.23		
Children (<18 years)											
Direct (medical and non-medical) ($)	$362.95	$119.11	$123.47				$59.34	$99.20	$43.69	$64.98	$8.17
Indirect (days of work/sick leave/school lost)	22.17	20.14	13.87								
Indirect cost of patients and caregivers ($)	$24.84	$11.53	$3.14			$172.65	$8.99	$17.40	$33.49	$13.00	$4.90
Study Characteristics											
Perspective	Patient and caregiver			Patient and provider	Patient	Societal	Patient and provider				
Type of enteric fever	Typhoid and paratyphoid			Typhoid	Typhoid	Typhoid	Typhoid				
Indirect costs	Only adults (≥18 years)			Working children and adults	Children and adults	Children and adults	Children and adults				

Data are presented in 2018 US dollars. Results from other countries were adjusted by inflating local currencies using local inflation rates and then exchanging to US dollars. The studies in this table have methodological differences that prevent them to be directly comparable. Source: references [7–11, 19].

^a^ Only includes children are <18 years and indirect costs corresponds exclusively to caregivers.

^b^ In this study children are ≤15 years.

^c^ In this study children are 5–17 years.

^d^ In this study children are 2–15 years.

### Limitations

This study has several limitations. Eligible patients included those with nontraumatic terminal ileal perforation, which sometimes follows acute enteric fever cases; although those with a known alternative diagnosis were excluded, it is possible that some might have been misclassified. In the patient and caregiver COI sample, 12.3% of patients had such perforations and negative blood culture results, providing a maximum bound on the potential inclusion of non–enteric fever patients in the main analysis with all patients. The patient and site samples are not representative of Pakistan, and only reflect patients who sought care at the sampled hospitals. Thus, care should be taken in generalizing results of this study beyond similar populations. Self-reported patient and caregiver costs may also be subject to recall or reporting bias. Patient and caregiver COI interviews were conducted by phone instead of in person, which could have affected the response rate. A control group to account for potential background patient morbidity and health care costs was not included. The study does not model the risk of increasing antimicrobial-resistant enteric fever and its associated costs.

Among the limitations of the health care provider COI is the imputation of some medical supply prices and administrative costs based on third-party sources (eg, other hospitals and UNICEF), which may have increased or decreased the estimated costs compared to the real prices. Some elements of the health care provider COI (eg, personnel time per procedure) may be subject to recall or reporting biases, which may over- or underestimate the COI.

Finally, costs were not combined across perspectives due to the limited health facility sample, which did not represent all health facilities visited by patients at which patient and caregiver costs were incurred, and the limited ability of patients and caregivers to recall and report expenses for specific clinical procedures (eg, by specific type of lab test) to match these with health facility costs.

## CONCLUSIONS

The COI estimates presented here illustrate that the economic burden of enteric fever is considerable to patients, their caregivers, and health care providers in Pakistan, especially for cases with complications, such as nontraumatic ileal perforation. In combination with other evidence on the disease burden and the costs and effectiveness of interventions, understanding the magnitude of the economic burden of enteric fever is important to assess the value of interventions aiming to control enteric fever, and to inform programmatic and strategic decisions around typhoid Vi-conjugate vaccine introduction strategies and improvements to water and sanitation in Pakistan and other endemic countries.

## Notes

***Acknowledgments.*** The authors thank the Surveillance for Enteric Fever in Asia Project (SEAP) Pakistan team, especially Irum Fatma, Hina Mirza, Najeeb Rahman, and Ashar Malik; personnel in the SEAP Pakistan study sites; personnel at the International Centre for Diarrhoeal Disease Research, Bangladesh; and other members of the SEAP project team who supported data collection and management, especially Ashley Tate Longley and Caitlin Barkume.

***Disclaimer.*** The results and conclusions in this article are those of the authors and do not necessarily represent the official position or policies of the US Centers for Disease Control and Prevention or other partners.

***Financial support.*** This work was supported by funding from the Bill and Melinda Gates Foundation (Investment ID grant number INV-008335) and the US Centers for Disease Control and Prevention.

***Supplement sponsorship.*** This supplement is sponsored by the Sabin Vaccine Institute and made possible by a grant from the Bill & Melinda Gates Foundation.

***Potential conflicts of interest.*** K. D., T. A., S. P., and N. M. report a conditional gift agreement between the Sabin Vaccine Institute (Primary grantee for the Surveillance for Enteric Fever in Asia Project grant from the Bill and Melinda Gates Foundation) and the CDC Foundation. All other authors report no potential conflicts. All authors have submitted the ICMJE Form for Disclosure of Potential Conflicts of Interest. Conflicts that the editors consider relevant to the content of the manuscript have been disclosed.

## References

[1] Global Burden of Disease 2017 Typhoid and Paratyphoid Collaborators. The global burden of typhoid and paratyphoid fevers: a systematic analysis for the Global Burden of Disease Study 2017. Lancet Infect Dis 2019; 19:369–81. 30792131 10.1016/S1473-3099(18)30685-6 PMC6437314

[2] Haddix AC, Teutsch SM, Corso PS. Prevention effectiveness: a guide to decision analysis and economic evaluation. New York, NY: Oxford University Press, 2003.

[3] World Health Organization. Antimicrobial resistance: global report on surveillance. Geneva, Switzerland: World Health Organization, 2014.

[4] World Bank. World development indicators. 2019 Available at: https://datacatalog.worldbank.org/dataset/world-development-indicators. Accessed 7 November 2019.

[5] Institute for Health Metrics and Evaluation. Global health data exchange. Global Burden of Disease study 2017. 2020 Available at: http://ghdx.healthdata.org/gbd-2017. Accessed 15 January 2020.

[6] Gavi The Vaccine Alliance. Pakistan becomes first country to introduce new typhoid vaccine into routine immunisation program. 2019 Available at: https://www.gavi.org/news/media-room/pakistan-becomes-first-country-introduce-new-typhoid-vaccine-routine-immunisation#:~:text=Pakistan%20becomes%20first%20country%20to%20introduce%20new%20typhoid,vaccine%20to%20offer%20protection%20against%20increasingly%20drug-resistant%20disease Accessed 25 February 2020.

[7] Poulos C, Riewpaiboon A, Stewart JF, et al Cost of illness due to typhoid fever in five Asian countries. Trop Med Int Health 2011; 16:314–23. 21223462 10.1111/j.1365-3156.2010.02711.x

[8] Bahl R, Sinha A, Poulos C, et al. Costs of illness due to typhoid fever in an Indian urban slum community: implications for vaccination policy. J Health Popul Nutr 2004; 22:304–10. 15609783

[9] Kaljee LM, Pach A, Garrett DO, Bajracharya D, Karki K, Khan I Social and economic burden associated with typhoid fever in Kathmandu and surrounding areas: a qualitative study. J Infect Dis 2017; 218:S243–9. 10.1093/infdis/jix122 PMC6226633 28973415

[10] Mejia N, Pallas SW, Saha S, et al. Typhoid and paratyphoid cost of illness in Bangladesh: patient and health facility costs from the Surveillance for Enteric Fever in Asia Project (SEAP) II. Clin Infect Dis 2020. 10.1093/cid/ciaa1334 PMC7750988 33258940

[11] Mejia N, Abimbola T, Andrews J, et al. Typhoid and paratyphoid cost of illness in Nepal: patient and health facility costs from the Surveillance for Enteric Fever in Asia Project (SEAP) II. Clin Infect Dis 2020. 10.1093/cid/ciaa1335 PMC7750979 33258938

[12] World Health Organization. Typhoid vaccines: WHO position paper–March 2018. Wkly Epidemiol Rec 2018; 13:153–72.

[13] Aguinis H, Gottfredson RK, Joo H Best-practice recommendations for defining, identifying, and handling outliers. Organ Res Methods 2013; 16:270–301.

[14] Employers Federation of Pakistan. Sindh minimum Wage Notification 8 October 2018. 2018 Available at: https://efp.org.pk/sindh-minimum-wage-notification-8-october-2018/ Accessed 28 October 2020.

[15] World Health Organization. WHO global observatory data repository–life expectancy by country. 2016 Available at: http://apps.who.int/gho/data/node.main.688?lang=en. Accessed 20 March 2020.

[16] Pakistan Bureau of Statistics. Consumer price index. 2019 Available at: http://www.sbp.org.pk/ecodata/MPM.pdf. Accessed 7 November 2019.

[17] National Bank of Pakistan. Exchange rates. 2019 Available at: https://www.nbp.com.pk/RateSheet/index.aspx. Accessed 7 November 2019.

[18] United Nations International Children’s Emergency Fund. Supply catalog. 2019 Available at: https://supply.unicef.org/. Accessed 15 October 2019.

[19] Riewpaiboon A, Piatti M, Ley B, et al. Cost of illness due to typhoid fever in Pemba, Zanzibar, East Africa. J Health Popul Nutr 2014; 32:377–85. 25395900 PMC4221443

[20] Xu K, Evans DB, Kawabata K, Zeramdini R, Klavus J, Murray CJ Household catastrophic health expenditure: a multicountry analysis. Lancet 2003; 362:111–7. 12867110 10.1016/S0140-6736(03)13861-5

[21] Xu K, Evans DB, Carrin G, Aguilar-Rivera AM, Musgrove P, Evans T Protecting households from catastrophic health spending. Health Aff (Millwood) 2007; 26:972–83. 17630440 10.1377/hlthaff.26.4.972

[22] Raban MZ, Dandona R, Dandona L Variations in catastrophic health expenditure estimates from household surveys in India. Bull World Health Organ 2013; 91:726–35. 24115796 10.2471/BLT.12.113100 PMC3791647

